# Differentiation and Selection of Hepatocyte Precursors in Suspension Spheroid Culture of Transgenic Murine Embryonic Stem Cells

**DOI:** 10.1371/journal.pone.0044912

**Published:** 2012-09-24

**Authors:** Elke Gabriel, Stephanie Schievenbusch, Eugen Kolossov, Jan G. Hengstler, Tamara Rotshteyn, Heribert Bohlen, Dirk Nierhoff, Jürgen Hescheler, Irina Drobinskaya

**Affiliations:** 1 Institute of Neurophysiology, Center of Physiology and Pathophysiology, University of Cologne, Cologne, Germany; 2 Gastroenterology and Hepatology Clinic, University of Cologne, Cologne, Germany; 3 Axiogenesis AG, Cologne, Germany; 4 Leibniz Research Centre for Working Environment and Human Factors (IfADo), Technical University of Dortmund, Dortmund, Germany; Instituto Butantan, Brazil

## Abstract

Embryonic stem cell-derived hepatocyte precursor cells represent a promising model for clinical transplantations to diseased livers, as well as for establishment of *in vitro* systems for drug metabolism and toxicology investigations. This study aimed to establish an *in vitro* culture system for scalable generation of hepatic progenitor cells. We used stable transgenic clones of murine embryonic stem cells possessing a reporter/selection vector, in which the enhanced green fluorescent protein- and puromycin *N*-acetyltransferase-coding genes are driven by a common alpha-fetoprotein gene promoter. This allowed for “live” monitoring and puromycin selection of the desired differentiating cell type possessing the activated alpha-fetoprotein gene. A rotary culture system was established, sequentially yielding initially partially selected hepatocyte lineage-committed cells, and finally, a highly purified cell population maintained as a dynamic suspension spheroid culture, which progressively developed the hepatic gene expression phenotype. The latter was confirmed by quantitative RT-PCR analysis, which showed a progressive up-regulation of hepatic genes during spheroid culture, indicating development of a mixed hepatocyte precursor-/fetal hepatocyte-like cell population. Adherent spheroids gave rise to advanced differentiated hepatocyte-like cells expressing hepatic proteins such as albumin, alpha-1-antitrypsin, cytokeratin 18, E-cadherin, and liver-specific organic anion transporter 1, as demonstrated by fluorescent immunostaining. A fraction of adherent cells was capable of glycogen storage and of reversible up-take of indocyanine green, demonstrating their hepatocyte-like functionality. Moreover, after transplantation of spheroids into the mouse liver, the spheroid-derived cells integrated into recipient. These results demonstrate that large-scale hepatocyte precursor-/hepatocyte-like cultures can be established for use in clinical trials, as well as in *in vitro* screening assays.

## Introduction

Establishment of a hepatocyte-like *in vitro* differentiation system is a topical issue because of the need for a reproducible and accessible tool that could be used in early drug discovery and toxicology screening. Also, this system could feasibly serve as a source of hepatocytes for clinical transplantations into the diseased liver. However, there are certain requirements which should be fulfilled to achieve these goals: (a) an unlimited source of initial cell material is needed to ensure a routine large-scale generation of the required cells; (b) the generated cultures should be reproducible in terms of their hepatic-like functionality; and (c) the established system should allow for a highly efficient selection of hepatocytes.

Once the suitable hepatocyte-like cell model has been developed, it will have undisputable advantages over primary hepatocytes or established transformed hepatic cell lines as, for example hepatoma cells [1]. Although transplantation of isolated hepatocytes has been confirmed to be a low invasive intervention resulting in support of liver function, it is limited by shortage of sources of hepatocytes and also by a low level of expansion of transplanted cells in the host liver [2]–[7]. In addition, primary hepatocytes rapidly dedifferentiate displaying a decline of liver-specific mRNAs (including those for many metabolic genes) and a progressive decrease of albumin secretion, suggesting loss of their functionality [8]–[11]. Hepatic cell lines also show large differences compared to the *in vivo* situation. For instance, several cell lines obtained from hepatomas, while secreting albumin, lack the majority of drug-metabolising enzymes [12]. This restricts the application of both as screening *in vitro* test models.

In contrast to primary hepatocytes or hepatic cell lines, embryonic stem cell (ESC)-derived hepatocytes could represent a convenient material for both clinical trials and drug and toxicology tests, since they can be unlimitedly sourced by renewable and pluripotent ESCs. Protocols for their generation can be standardized, thus ensuring a high reproducibility of their functional characteristics. Although there are numerous studies on *in vitro* differentiation of hepatocytes from murine or human ESCs, in which certain hepatic features of the generated cultures have been assessed [13]–[19], the main associated problems have not yet been solved, such as a relatively low yield of differentiated cells, heterogeneity of resulting cultures, as well as insufficient selection of target cells. The latter issue is especially important in terms of complete elimination of undifferentiated ESCs possessing a high risk of teratoma formation after autologous transplantation of the cultured cells or after their transplantation into immunosuppressed recipients [20], [21].

A process leading to development of hepatocytes from the definitive endoderm of the ventral foregut begins at approximately embryonic day 8.5 (E8.5) in the mouse. Cardiac mesoderm signalling specifies the definitive endoderm to adopt a hepatic fate. Subsequent outgrowth of the hepatic endoderm, induced by the contact between the endoderm and the septum transversum mesenchyme results in the formation of the liver bud by E9.5, followed by proliferation and maturation of the liver throughout embryogenesis (for Review, see [22], [23]). ESC aggregates (“embryoid bodies” (EBs)) are capable of differentiating into all three germ layers including the endoderm, mesoderm, and ectoderm [24], imitating the early steps of embryonic development. Cells of the outer rim of EBs were shown to resemble endodermal cells in regard to the expression of endodermal markers including alpha-fetoprotein (Afp) [25]–[28]. The hepatocyte-like differentiation of the outer-layer Afp-expressing cells after their separation from EBs has been demonstrated [28]–[30].

We previously generated hepatocyte precursor- and hepatocyte-like cultures, using murine transgenic ESC clones that enable “live” monitoring and antibiotic selection of differentiating cells [31]. That study showed that the hepatocyte-committed cell fraction, which developed in the outer layer of EBs cultured in suspension, was able to form spheroids comprising proliferating cells. Once plated onto an adhesive matrix, the spheroids formed outgrowing colonies. This behaviour was similar to that of liver progenitor/stem cells [32], and it was therefore of great interest to further investigate the spheroid cultures for their hepatocyte precursor features and, correspondingly, for their ability to differentiate into functional hepatocyte-like cells.

Another important issue to consider is the scale-up of hepatocyte-like cultures, especially in view of their application to clinical trials and *in vitro* screening. Currently, there are no approaches available that allow for a large-scale generation of purified hepatocyte-like cell populations. In the present study, we established a scalable dynamic cell system based on a mass culture of transgenic mouse ESCs, enabling production of highly purified hepatocyte precursor cells capable of further differentiating into hepatocytes.

## Materials and Methods

### Ethics Statement

All animal experimental procedures were approved by the State Office for Nature, Environment and Consumer Protection of North-Rhine Westphalia, Germany (LANUV NRW reference number 8.87-50.10.37.09.279) and were conducted under protocols approved by the Animal Care Use Committee of the University of Cologne and were in accordance with US National Institutes of Health Guidelines.

### Transgenic ESC clones

**Figure 1:**
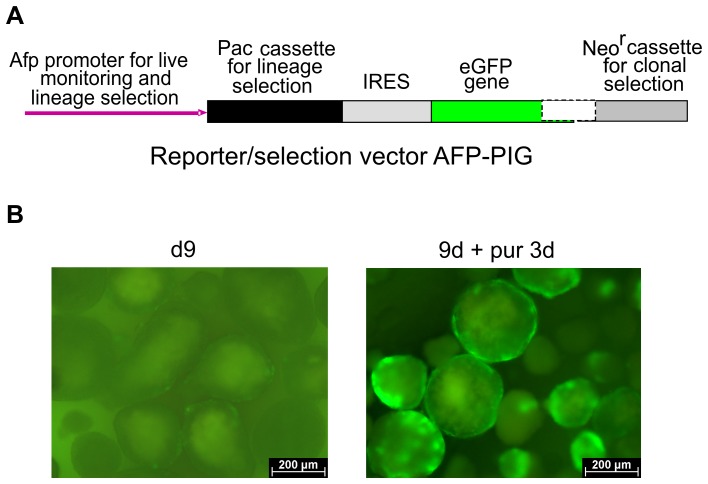
Differentiation and pre-selection of eGFP-expressing cells in a rotary EB culture. (**A**) Essential part of the AFP-PIG vector (“PIG”: after Pac-IRES-eGFP). (**B**) eGFP-positive cells in the outer rim of 9-day-old EBs (left panel) and appearance of expanding clusters of eGFP-expressing cells after a 3-day treatment with puromycin (right panel).

The design of the reporter/selection vector called AFP-PIG (see schematic drawing in Figure 1A) and the generation of stable transgenic ESC clones possessing this vector have been previously reported on in detail [31]. The vector contains the Afp gene promoter-enhancer driving both enhanced green fluorescent protein (eGFP) and puromycin *N*-acetyltransferase (Pac) genes connected via the Internal Ribosomal Entry Site (IRES) ensuring a separate translation of the corresponding proteins from a common mRNA. Briefly, the transgenic clones were produced by transfection of the vector into mouse ESCs of the line D3 [24] by electroporation. The transgenic cells or clones are hereafter referred to as “AFP-PIG ESCs” or “AFP-PIG clones”. The results outlined below are generated from AFP-PIG clone 11, which has been used previously [31].

### Cultivation of AFP-PIG ESCs

AFP-PIG ESCs were stored in liquid nitrogen and then thawed and seeded onto a feeder layer of mitomycin-treated mouse embryonic fibroblasts for two days in Dulbecco's modified Eagle's medium (DMEM) containing 15% (v/v) fetal bovine serum (FBS), 0.1 mM MEM non-essential amino acids, 100 U/ml penicillin, 100 µg/ml streptomycin (all from Invitrogen, Eggenstein, Germany), 0.05 mM β-mercaptoethanol (Sigma-Aldrich, München, Germany), 500 U/ml leukemia inhibitory factor (LIF, ESGRO) (Chemicon International, Hampshire, UK), und 300 µg/ml G418 (Invitrogen). The culture underwent two further passages on the feeder layer in the above-described medium, according to the follows scheme: cells were seeded at a density of 11,000 cells per cm^2^ and cultured for three days, and in the subsequent passage the cells seeded at 18,000 cells per cm^2^ were cultured for additional two days. Cell colonies were dissociated with 0.25% trypsin-EDTA (Invitrogen) immediately before seeding.

### Generation of EB mass culture. Monitoring and initial antibiotic selection of an eGFP-expressing cell fraction

AFP-PIG ESC colonies were dissociated with 0.25% trypsin-EDTA. The single cell suspension containing 2×10^6^ cells in 10 ml of DMEM was transferred to bacterial Petri dishes (Greiner Bio-One, Frickenhausen, Germany) placed on a shaker (reciprocating/20 mm/93 min^−1^) and incubated at 37°C and 5% CO_2_. From this step of the differentiation protocol onward, DMEM (referred to in the sections below as “medium”) contained neither LIF nor G418 but was supplied with 20% (v/v) FBS. After 2 days EB formation on the shaker, a spinner flask (SF) EB culture was set up. 500-ml SFs with duopendulums (EBS Integra Bioscience, Ruhberg, Germany) were coated with sigmacote (Sigma-Aldrich) according to the manufacturer's protocol. EBs (25,000) in 250 ml medium per SF (four SFs in total) were maintained on a rotary magnet platform of the Cellspin Spinner system (EBS Integra Bioscience) at 37°C and 5% CO_2_ for 7 days. The culture was rotated in alternating directions (4 rotations per direction) at 35 rpm. On day 3 after starting the SF culture, the culture medium was completely replaced with fresh medium. Three days later, half of the medium was changed, and a final complete medium change was performed on day 7. This time point was referred to as “day 9” of the EB mass culture, since it takes into account the initial 2 day culture on the shaker. Accordingly, EBs at this protocol stage were termed as “9-day-old EBs”. To prevent hematopoietic differentiation (see “Results”), the medium was supplied with 0.7 µg/ml rat tail collagen Type I (Invitrogen) for the entire 7-day-long EB maintenance in SFs. The EBs were then exposed to puromycin (Sigma-Aldrich) (5 µg/ml) for additional 3 days (the same puromycin concentration was used throughout the investigations described here). eGFP-fluorescent cells within EBs were monitored in a live mode under the fluorescent microscope Axio Observer, using the Axio Vision 4.8 software (both from Carl Zeiss MicroImaging, Jena, Germany) and the HQ-Longpass Filterset for Enhanced-GFP (AHF Analysentechnik, Tübingen, Germany). All microscopic observations described were performed using the same microscope and software; all filter sets used were from AHF.

To generate a reference static EB culture, the EBs formed during the shaker procedure were maintained as described previously [31]. Briefly, the culture was diluted to approximately 30 EBs per 1 ml medium and then incubated in square Petri dishes without shaking. Half of the medium was changed every 2 days. Nine-day-old EBs were concentrated to about 150 EBs per 1 ml medium and exposed to puromycin for 3 additional days. The culture was observed for development of eGFP-expressing cells as described above.

### Spheroid culture procedure. Further puromycin selection of eGFP-expressing cells

After the 3-day exposure of EBs to puromycin, the SFs were removed from the magnet platform and, after the EBs sedimented over several minutes, the medium was aspirated until 50 ml remained per SF. The EB suspension samples were then transferred to 50-ml Falcon tubes and centrifuged at 1000 rpm for 3 minutes. After aspiration of the supernatant, each pellet was re-suspended in 8 ml of pre-warmed 0.1% Worthington collagenase Type 2 solution (Cell Systems, St. Katharinen, Germany) in phosphate buffered saline (PBS) (Invitrogen) containing 0.02 mM CaCl_2_ and 80 mM MgCl_2_. The samples were incubated on a shaker (reciprocating/20 mm/150 min^−1^) at 37°C for 10 minutes, gently re-suspended, and 42 ml of medium per sample was added to dilute the enzyme. The cells were then centrifuged at 1000 rpm for 4 minutes, re-suspended in 10 ml medium and filtered through 100-µm pore nylon cell strainers (BD Biosciences, Heidelberg, Germany) to remove any non-dissociated EBs. Cell aggregates passing through the filters (referred to as “0-day-old spheroids”) were placed in SFs filled with 100 ml medium each. The culture was then rotated in a single direction at a velocity of 25 rpm at 37°C and 5% CO_2_. One day after starting the spheroid SF procedure, puromycin was added to the medium. Spheroid formation and growth were monitored by light and fluorescent microscopy. A reference static EB culture was treated with collagenase and filtered through 100-µm pore cell strainers. The resulting cell aggregates were maintained in Petri dishes without shaking and treated with puromycin in the same way as the spheroid culture in SFs. For quantitative estimation of spheroid growth, the diameter of 50 cell aggregates/spheroids was measured for each culture type on day 0 and then on days 1, 2, and 3 of the spheroid culture, using the Axio Vision 4.8 software. The respective average spheroid diameters were calculated. Statistical evaluation was performed by means of data of three independent cultures maintained in spinner flasks or in Petri dishes, using IBM® SPSS software, version 20. Student's unpaired t-test was performed, and values of p<0.05 have been considered to indicate statistical significance.

### Generation and monitoring of adherent spheroid-derived cultures

Two-day-old spheroids were plated onto cell culture plates coated with rat tail collagen Type I (Invitrogen) monolayer according to the manufacturer's protocol. The culture was maintained for 2 days in puromycin-containing medium at 37°C and 5% CO_2_. The drug was then washed out and the adherent culture was maintained further under the same conditions for various time periods. The morphology of outgrowing colonies was continuously observed by light microscopy, and eGFP fluorescence was monitored using the HQ-Longpass Filterset for Enhanced-GFP.

### Isolation and culture of primary mouse hepatocytes

Primary hepatocytes were isolated from 8 to 12 week old male C57BL6/N mice (Charles River, Sulzfeld, Germany), using a collagenase Type 2 perfusion method, and maintained on collagen Type I-coated plates as described by Godoy et al. [10]. Briefly, isolated hepatocytes were plated in a 12-well culture plate at a density of 4×10^4^ cells/cm^2^ in Williams' medium E (Pan-Biotech, Aidenbach, Germany) supplied with 2 mM L-glutamine, 100 U/ml penicillin, 100 µg/ml streptomycin, 10% (v/v) FBS (Invitrogen), and 100 nM dexamethasone (Sigma-Aldrich). Four hours after attachment, the cells were washed three times with Hank's Balanced Salt Solution (HBBS) (Invitrogen) and incubated overnight in serum-free Williams' medium E containing L-glutamine, penicillin, streptomycin, and dexamethasone.

### Quantitative real-time polymerase chain reaction

Total RNA was extracted from ESCs, eGFP-expressing cell aggregates immediately after their separation from EBs by collagenase treatment (0-day-old spheroids), from 2-day-old spheroid cultures and 12-day-old spheroid-derived adherent cultures, and from fetal (E14) and adult (10 weeks old) liver. RNeasy Plus Micro Kit (QIAGEN, Hilden, Germany) was used for RNA isolation (including DNase treatment) according to the manufacturer's instructions. Four separate RNA isolations were performed for each type of cell culture and for both reference liver tissues. RNA samples were analysed for expression of the following genes: forkhead box A2 (*Foxa2*), alpha-fetoprotein (*Afp*), albumin (*Alb*), liver-specific organic anion transporter 1 (*lst-1*), tyrosine aminotransferase (*Tat*), cytochrome P450, family 7, subfamily a, polypeptide 1 (*Cyp7A1*), and multidrug resistance-associated protein 2 (*Mrp2*). RNA was reverse-transcripted with the Moloney murine leukaemia virus reverse transcriptase (Sigma-Aldrich) and random hexamer primers (QIAGEN). Real-time quantitative PCR (qRT-PCR) was performed using Applied Biosystems' Real-Time PCR 7500 Fast System (Life Technologies, Darmstadt, Germany). Triplicates of 10 µl samples contained 5 µl TaqMan® Fast Advanced Master Mix, 0.5 µl TaqMan® Gene Expression Assays (both from Life Technologies), and a cDNA volume corresponding to 100 ng of the RNA template per sample. The thermal cycling profile was as follows: polymerase activation (Holding Stage) at 95°C for 20 seconds followed by 40 cycles of 95°C for 3 seconds and 60°C for 30 seconds. Threshold cycle numbers (C_T_) measured were normalized to those for hypoxanthine guanine phosphoribosyl transferase (*Hprt*) and TATA box binding protein (*Tbp*) as reference genes. PCR data were analysed using the 2^−ΔCT^ method [33] and their statistical evaluation was performed with help of IBM® SPSS software, version 20. The Mann-Whitney-U test for unpaired samples was carried out and values of p<0.05 were used as a criterion of statistical significance.

Alternative gene designations and their Entrez Gen data base IDs, as well as corresponding TaqMan® Gene Expression Assays are listed in Table S1.

### Fluorescent immunoassay for marker proteins

Spheroid-derived 5-day- and 12-day-old adherent cultures and primary hepatocytes were washed with PBS containing 0.9 mM Ca^2+^ and 0.5 mM Mg^2+^ and fixed with 4% paraformaldehyde (PFA) in PBS (the 16% stock PFA solution was purchased from Polysciences Europe, Eppelheim, Germany). After washing with PBS, cells were permeabilized for 10 minutes in a solution of 0.5 M NH_4_Cl and 0.25% Triton X-100 (both from Applichem, Darmstadt, Germany) in PBS. Cultures were then washed with PBS and treated with Image-iT® FX signal enhancer (Invitrogen) for 30 minutes at room temperature to quench autofluorescence. Non-specific binding sites were blocked with 1∶10 Roti®-ImmunoBlock (Carl Roth, Karlsruhe, Germany)/H_2_O solution for 1 hour at room temperature. Afterwards, the samples were incubated with primary antibodies overnight at 4°C: goat polyclonal anti-Alb (albumin) IgG (1∶50), goat polyclonal anti-Aat (alpha-1-antitrypsin) IgG (1∶50), goat polyclonal anti-CK-18 (cytokeratin-18) IgG (1∶100), goat polyclonal anti-Ecad (E-cadherin) IgG (1∶100), and goat polyclonal anti-lst-1 (liver-specific organic anion transporter 1) IgG (1∶100). All primary antibodies used were from Santa Cruz Biotechnology (Heidelberg, Germany). The antibody dilutions were prepared in a Roti®-ImmunoBlock/PBS (1∶100) solution. After washing out the primary antibodies, Alexa Fluor® 555-conjugated donkey anti-goat IgG (Invitrogen) diluted 1∶1000 in PBS was added for 1 hour at room temperature in darkness. The cultures were then washed with PBS and stained with Hoechst 33342 for cell nuclei for 7 minutes at 37°C. Alexa Fluor® 555 fluorescence was observed using the HQ-Filterset for Cy3 and nuclei were detected with the Filterset for DAPI, Hoechst.

Cryosections of liver tissue with a thickness of 5 µm were prepared as described below (see “Transplantation of spheroids and monitoring of intrahepatic engraftment”). The cryosections were then transferred to glass object slides, air-dried and rehydrated with PBS. Non-specific binding sites were blocked with 5% donkey serum (Sigma-Aldrich), and the sections were then incubated with monoclonal rat anti-Ecad antibody (TaKaRa, Saint-Germain-en-Laye, France) (1∶100) at 37°C for 1 hour. After washing with 0.05% Tween 20 in PBS, the primary antibody was detected by application of Cy3-conjugated donkey anti-rat IgG (Dianova, Hamburg, Germany) (1∶50) at 37°C for 1 hour in darkness. Cell nuclei were visualized by Hoechst 33342 staining. Cy3 fluorescence was detected using the HQ-Filterset for Cy3, and nuclei were observed with the Filterset for DAPI, Hoechst.

Alternative designations of proteins analysed and their UniProt data base IDs are listed in Table S2.

### Detection of glycogen storage in spheroid-derived adherent cultures

Adherent spheroid-derived cultures were fixed with 4% PFA, washed twice with distilled water and then stained for glycogen using the Periodic acid-Schiff Kit (Sigma-Aldrich) according to manufacturer's instructions. After washing with PBS, cultures were counterstained with hematoxylin. Observations were carried out using light microscopy.

### Monitoring of indocyanine green up-take and release in adherent cultures

Indocyanine green (ICG) (Sigma-Aldrich, defined as “Cardiogreen”) was dissolved to 25 mg/ml in DMSO and then diluted in DMEM to 1 mg/ml. After aspiration of medium in wells containing spheroid-derived colonies, the ICG/DMEM solution was added for 1 hour at 37°C and 5% CO_2_. ICG was then removed by washing with medium, and light microscopy images were taken immediately. The cultures were next incubated in medium for additional 6 hours under the same conditions as before. Light microscopy observation for ICG release was then carried out.

### Detection of undifferentiated ESCs in EB and spheroid cultures

Puromycin-treated 9-day-old EBs and 2-day-old spheroids were tested for the presence of undifferentiated ESCs using the “feeder test”, as described previously [31], [34]. Briefly, the cultures were dissociated with TrypLE™ Select (Invitrogen), washed once with medium, re-suspended and seeded onto a fibroblast feeder layer at a density of 10,000 cells per cm^2^ (200,000 cells in total) for 2 weeks under conditions typically used for propagation of ESCs. The cultures were microscopically monitored for the appearance of ESC colonies.

### Transplantation of spheroids and monitoring of intrahepatic engraftment

Male 129S2/SvPasCrl wild type mice were purchased from Charles River (Sulzfeld, Germany). The animals were housed at a constant temperature (22°C–23°C) with a 12 hour light-dark cycle and free access to regular chow and water. Six-week-old mice were treated with the DNA-alkylating reagent retrorsine (Sigma-Aldrich) (30 mg/kg body weight, intraperitoneally) 4 and 2 weeks prior to transplantation to impair hepatocyte proliferation in the recipients' liver. For transplantation, animals were anesthetized using carprofen (Pfizer, Berlin, Germany) (50 mg/kg body weight) and isoflurane (DeltaSelect, Dreieich, Germany). After 1/3 partial hepatectomy, spheroids that had been suspended in 200 µl medium (approximately 0.5×10^6^ cells per sample) and then partially dispersed using a 200 µl pipette were injected into the spleen. Sham-operated mice receiving medium alone served as negative controls. Mice were sacrificed 1 or 2 weeks after cell transplantation. Dissected livers were fixed with 4% neutral-buffered formaline (Carl Roth) for 4 hours and then embedded in 30% sucrose (Sigma-Aldrich) overnight. Finally, the liver tissue was snap frozen in liquid nitrogen and stored at −80°C. Cryosections with a thickness of 5 µm were microscopically evaluated for engraftment of eGFP-positive cells, using the HQ-Longpass Filterset for Enhanced-GFP.

## Results

### Differentiation and pre-selection of hepatocyte-committed cells in dynamic EB cultures

In this study we used stable transgenic mouse ESC-derived cell clones possessing the reporter/selection vector AFP-PIG, in which eGFP and puromycin-resistance (Pac) genes are driven by a common Afp gene promoter-enhancer. Since Afp is expressed in embryonic endoderm and, at a high level, in fetal hepatocytes from the early stages of liver embryogenesis onward [35], [36], the use of the transgenic AFP-PIG cell clones enabled the tracking of their hepatic lineage differentiation from the on-set of *Afp* expression through to later development stages.

In order to efficiently generate hepatocyte-committed cells possessing eGFP and puromycin resistance, we established an SF EB culture following the initial EB formation in a suspension mass culture of AFP-PIG ESCs on a shaker. The number of EBs on day 2 of the shaker culture was typically 40,000–60,000, derived from 2×10^6^ original ESCs placed in 10 ml of medium in a Petri dish (estimated from 13 experiments). Aliquots of 25,000 EBs were transferred to each SF for further culture. We discovered that the EBs were highly sensitive to culture conditions in SFs, which significantly complicated the establishment of a suitable protocol. We tested various culture parameters, such as medium volume per SF, EB density, mono- or bidirectional rotation mode, and rotation velocity. Although EBs grew in SFs and eGFP-positive cells were already evident by day 5, we frequently observed the appearance of EBs containing reddened cell areas. This is in line with the data suggesting stimulation of differentiation of mesoderm-derived haematopoietic-like cells in a three-dimensional spinner culture of mouse ESCs [37]. In this case, further enrichment of the culture with the eGFP-expressing cell fraction failed. Addition of 0.7 µg/ml collagen Type I to culture medium from the first day of the SF protocol for the remainder of the culture period, combined with the optimized rotation regime described in “Materials and Methods”, prevented the development of the presumably haematopoietic cells and caused a visual increase in the number and fluorescence intensity of eGFP-positive cells within EBs.

By day 7 of the SF culture (equivalent to 9-day-old-EBs, see “Materials and Methods”), the percentage of EBs containing eGFP-expressing cells predominantly in their outer rim (Figure 1B, left panel) typically reached 60% to 90% of the total EB number. This was similar to the number of eGFP-positive EBs cultured in Petri dishes, estimated in our previous study [31] and reproduced again in the reference static EB culture during this study; no EB loss was observed (data obtained on 4 cultures). Also, average sizes of 9-day-old EBs in the dynamic and static cultures were similar. EBs in SFs were somewhat more uniform in terms of their size and did not form clumps as it was with some EBs in Petri dishes. After puromycin treatment of EBs for additional 3 days, eGFP-fluorescent cell clusters abruptly expanded in the background of massively dying eGFP-negative cells (Figure 1B, right panel). Death of cells not expressing eGFP has been confirmed in our previous experiments by treatment of EBs with propidium iodide [31]. To assess the efficiency of the selection procedure at the EB protocol step (referred to as “pre-selection”), we carried out the “feeder test”. Two weeks after seeding 200,000 EB-derived cells on a fibroblast feeder layer, 120 to 370 ESC colonies were detected (data from 3 independent EB cultures), revealing the presence of 0.06–0.19% undifferentiated ESCs in the cell samples seeded. This suggested an effective elimination of undifferentiated ESCs in the course of the pre-selection procedure.

### Culture of selected eGFP-expressing cells, growing as spheroids in spinner flasks

**Figure 2:**
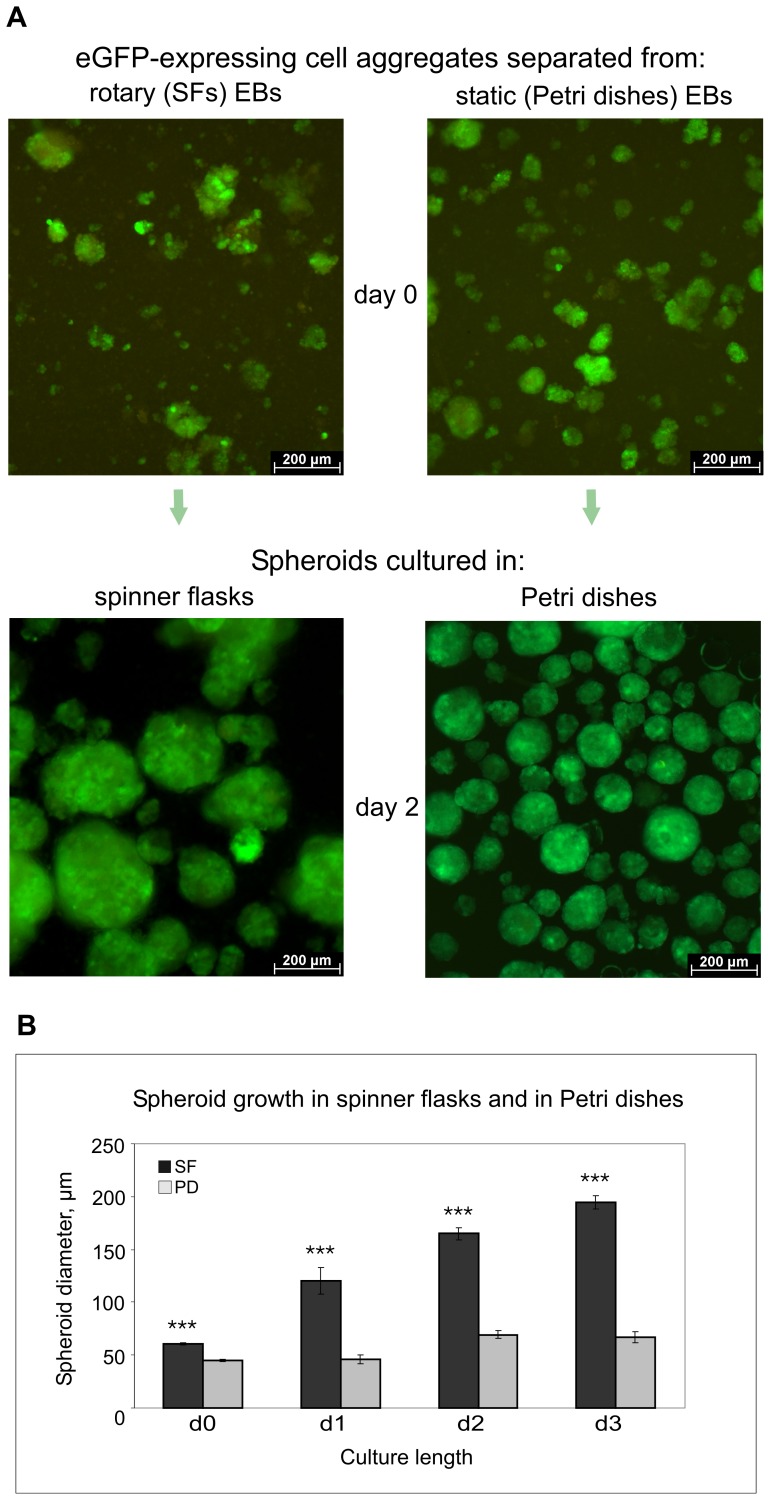
Spheroid formation and growth in dynamic and static conditions. (**A**) Top panels: eGFP-expressing cell aggregates immediately after their separation from EBs cultured in a rotary or in a static condition. Bottom panels: rotary and static cultures of 2-day-old spheroids. (**B**) Comparative diagrams of spheroid growth in SFs and in Petri dishes. The mean of the spheroid diameter ± standard error of the mean (SEM) are plotted. The sign (***) indicates extremely statistically significant differences (p<0.001) in spheroid size between the dynamic and static cultures.

To generate hepatic progenitor cells, we established an optimal method for collagenase treatment of EBs after their exposure to puromycin, as well as an appropriate protocol for generating and culturing spheroids in SFs. After collagenase separation of eGFP-expressing cell clusters from EBs, the resulting cell aggregates were maintained in SFs in the presence of puromycin. Microscopic observations revealed that spheroid grew over time in culture (Figure 2A), suggesting that cells proliferated within the spheroids. The diameters of spheroids maintained in SFs and, for comparison, in Petri dishes (static culture) were measured on day 0 (i.e., eGFP-expressing cell aggregates immediately after their separation from EBs) and then on days 1, 2, and 3 of the culture. We observed that spheroids in SFs possessed a significantly higher growth capacity than those in Petri dishes: on day 2, the average diameter of “dynamic” spheroids exceeded 2-fold that of “static” spheroids (see corresponding diagrams in Figure 2B). Many spheroids measured 300–400 µm in diameter and were comparable to 9-day-old EBs.

The total number of spheroids produced from one culture typically ranged from 2,000 to 3,000 (counted for 5 cultures). Taking into account that 4×10^6^ ESCs were required to generate 100,000 EBs for 1L EB culture in 4 SFs, the output of spheroids was 500 to 800 per 1×10^6^ original AFP-PIG ESCs. The same quantity of ESCs yielded in static condition 150 to 200 spheroids (data from 6 cultures), indicating approximately 2.5 to 5 fold less output. Given that spheroids in SFs were, in average, about 2-fold larger than those in Petri dishes, this suggested a 5 to 10 fold higher final yield of spheroid cells displaying features of hepatocyte precursors (see below) in dynamic spheroid cultures as compared to static ones.

To quantify the efficacy of puromycin selection/purification procedure, we disassembled 2-day-old spheroids with TrypLE™ Select and seeded the resulting single cell suspension (200,000 cells in total) onto a fibroblast feeder layer in the same way as for puromycin-treated EBs. Two weeks after seeding, only 1 to 6 ESC colonies were observed (data from three spheroid cultures), equating to 0.0005–0.003% ESCs that were present in the cell suspension seeded. When compared with the result of the “feeder test” on EBs treated with puromycin for 3 days (see the section above), this shows that the exposure of spheroids to puromycin resulted in a further high-degree selection/purification of the target culture.

### Gene expression profile of spheroid cultures

Based on eGFP expression and on the proliferation behaviour of cells within spheroids, together with our preliminary data on the expression of several hepatic marker genes in spheroid cultures (data not published), we assumed that these cells represent a population of hepatocyte precursors. To confirm this, we carried out RNA analysis of 0-day- and 2-day-old spheroids by qRT-PCR. For comparison, RNA isolated from ESCs and from fetal and adult liver was analysed.

**Figure 3:**
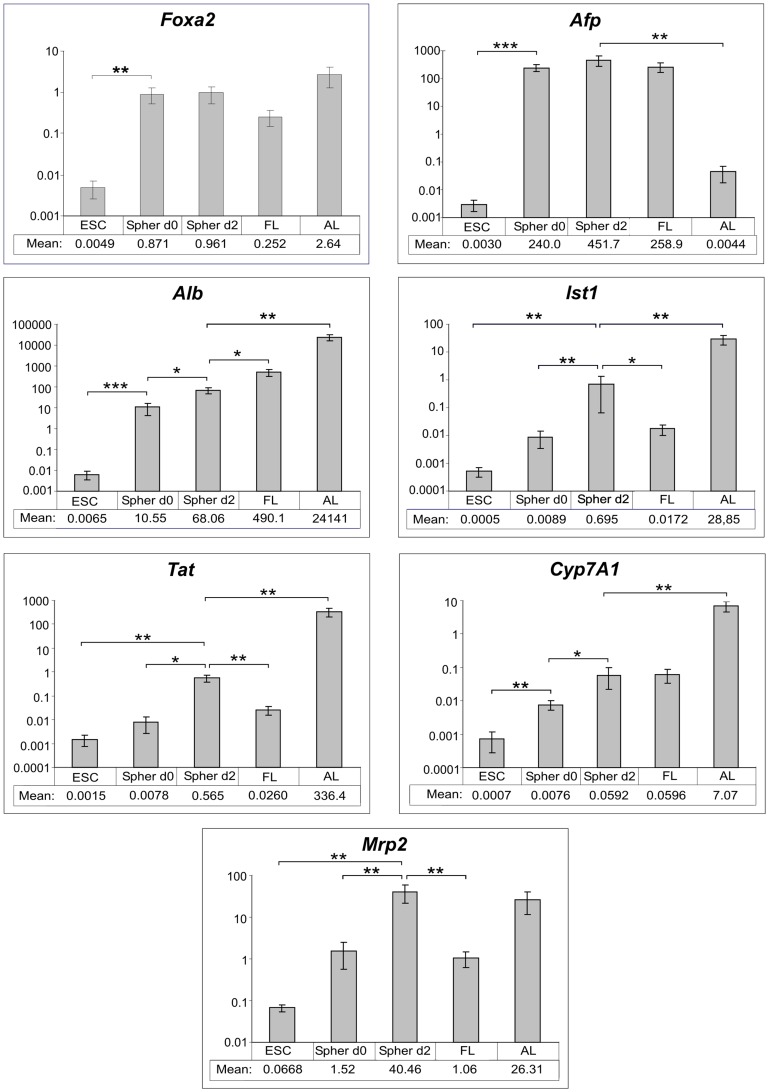
Gene expression profiles of dynamic spheroid cultures, ESCs, and fetal and adult liver tissue. Due to a big difference between expression values of single genes in particular culture and tissue types, qRT-PCR data are displayed for each gene separately using a logarithmic scale on the y-axes. Mean relative gene expression values ± standard error of the mean (SEM) are plotted. For a better overview, the mean values are additionally listed in tables below the diagrams. Abbreviations ESC, Spher d0, Spher d2, FL, and AL refer to undifferentiated ESCs, 0-day-old and 2-day-old spheroids and to fetal and adult liver, respectively. Statistical significance of the PCR data was evaluated using IBM® SPSS software. Symbols (*), (**) or (***) depict a statistically significant (p<0.05), a highly statistically significant (p<0.01) or an extremely statistically significant (p<0.001) difference in gene expression values, respectively. In control reverse transcription-negative samples and in none-template-containing blank samples, no PCR products have been detected (data not shown).

As shown in Figure 3, expression of *Foxa2*, which is required for determination of the definitive endoderm and maintenance of the endodermal lineage and also for gene regulation in hepatocytes [38]–[41], was already strongly up-regulated in eGFP-positive cell aggregates (i.e., 0-day-old spheroids) immediately separated from puromycin-treated EBs. The expression level remained unchanged by day 2 of the spheroid culture and was comparable to that in fetal and in adult liver. In 0-day-old spheroids, *Afp* expression was markedly higher (approximately 80,000-fold) than in ESCs but was of the same level as that in fetal liver cells. The *Afp* transcription value remained constant during further 2 days of culture. These data again confirmed the hepatocyte lineage commitment of the eGFP-positive cell fraction isolated from puromycin-treated EBs.

In 0-day-old spheroids, the gene coding for Alb, the main secreted liver protein [42], [43], was expressed at a much higher level than that in ESC cultures and was further up-regulated by day 2 of the spheroid procedure. The transcription of this gene in 2-day-old spheroids was about 7-fold lower than that in fetal liver. The difference between the *Alb* expression level in spheroid culture on day 2 and in adult liver was much more pronounced (approximately 360-fold). The genes encoding the anion transporter lst-1 specifically synthesized in hepatocytes and responsible for the majority of hepatic uptake of chemicals [44], [45] and the mature hepatocyte marker, Tat [46]–[48], were only marginally up-regulated before the start of the spheroid culture (day 0). After 2 days of the culture, expression of both genes was increased by approximately 70 fold, the levels being higher than those in fetal liver (approximately 40-fold and 20-fold for *lst-1*and *Tat*, respectively), but still remained at lower levels than in adult liver. Expression of the liver-specific cytochrome Cyp7A1 gene (with low expression in fetal liver but high expression in adult hepatic cells [46]) in eGFP-positive cell aggregates, which had been immediately separated from puromycin-treated EBs, was approximately 10% of the level in fetal liver. By day 2 of the spheroid culture, the *Cyp7A1* expression increased to the same level as measured in fetal liver tissue. We also analysed the gene expression of Mrp2, the member of the multidrug resistance protein subfamily, localized exclusively to the apical (canalicular) membrane domain of polarized cells, such as hepatocytes (for Review, see [49]). Mrp2 plays an important role in the elimination of phase II biotransformation products from hepatocytes into bile and its expression is much stronger in adult than in fetal liver [50]. The *Mrp2* expression in spheroids appeared to be activated over 2 days and exceeded the level in ESCs by approximately 600 fold. Moreover, the level of the *Mrp2* expression in 2-day-old spheroids reached a value very similar to that in adult liver.

These data indicate that the hepatic lineage-committed cell population, which had been selected from the rotary EB cultures, progressively developed the hepatic gene expression phenotype when maintained as a dynamic suspension spheroid culture. The fact that, on the one hand, the *Foxa2*, *Afp*, and *Cyp7A1* expression in spheroid cultures was essentially equal to that in fetal liver and, on the other hand, genes representing advanced differentiated hepatocytes, such as *Alb*, *lst-1*, and *Tat*, were significantly up-regulated after 2 days spheroid culture but remained expressed at lower levels than in adult liver tissue, suggests that the spheroid culture represented a hepatocyte precursor cell population likely comprising a fraction of fetal liver-like cells. The apparent activation of *Mrp2* expression in spheroids (almost to levels in adult liver) might be a sign of the appearance of advanced hepatocyte-like cells.

### Development of hepatocyte-like cells in spheroid-derived adherent cultures

**Figure 4:**
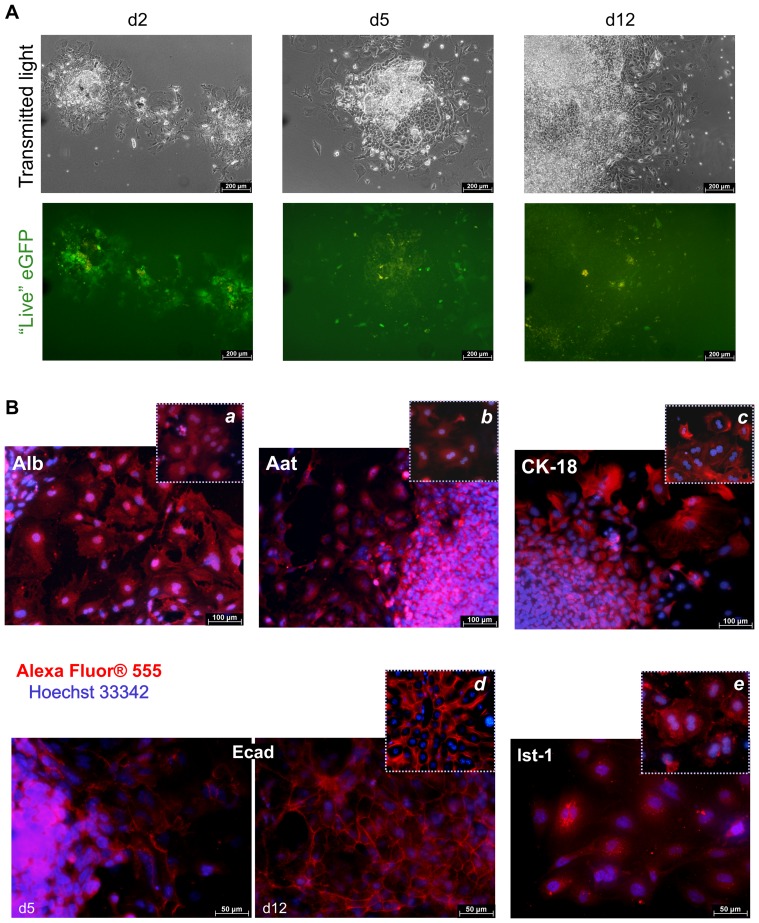
Expression of eGFP and hepatic proteins in spheroid-derived adherent cultures. (**A**) Cell colonies outgrowing on collagen Type I matrix. Down-regulation of eGFP fluorescence during the course of the colony growth is evident. (**B**) Expression of hepatic proteins, using fluorescent immunoassay. For better view of Ecad and lst-1 expression patterns across the cells, the corresponding images are displayed at a magnification ×400. Respective staining of primary hepatocytes (panels *a–c* and *e*) or of a liver tissue section (panel *d*) are shown for comparison. Alexa Fluor® 555-conjugated IgG served as a secondary antibody; cell nuclei were visualized by Hoechst 33342 staining. Merged Alexa Fluor® 555/Hoechst 33342 images are shown. Samples treated with an isotype control antibody instead of the primary antibody stained negatively (data not shown).

To investigate whether the cells generated and selected in spheroid cultures possessed a capability to differentiate into hepatocyte-like cells, we plated 2-day-old spheroids onto collagen Type I-coated plates. During the course of subsequent maintenance on the collagen matrix, spheroids formed expanding colonies (Figure 4A), again confirming the proliferation behaviour of spheroid-derived cells. eGFP fluorescence decreased as the colonies grew, temporary correlating with down-regulation of puromycin resistance. Thus, a prolonged exposure of the adherent culture to puromycin led to a massive death of eGFP-negative cells from day 3 to day 4 of the culture onward. As both the eGFP gene and the puromycin resistance cassette are driven by the common Afp gene promoter, this observation suggested down-regulation of the Afp gene activity, most probably reflecting a gradual progress of cell maturation. This conclusion is supported by the data on qRT-PCR analysis of 12-day-old spheroid-derived adherent cultures, which revealed a strong (about 450-fold, p = 0.02) down-regulation of *Afp* expression as compared to 2-day-old spheroids (data from 3 cultures, not shown).

We analysed expression of hepatic proteins in adherent cultures by fluorescent immunostaining. As shown in Figure 4B, as early as 5 days after plating spheroids, many cells within the colonies stained positively for Alb, Aat (hepatic positive acute-phase protein [51], [52]) and CK-18, the main Type I cytokeratin in differentiating hepatic cells [53], [54]. Cytoplasm proteins, Alb and Aat, were diffusely spread across the cells (with a higher protein density around cell nuclei in the case of Aat), and CK-18 staining was of a typical intermediate filament pattern. Also, expression of the general adhesion marker protein of epithelial cells, Ecad [55], [56], which is synthesized in liver biliary cells and periportal hepatocytes [56] and also in liver stem cells [57], was already detectable in 5-day-old colonies (see the left Ecad panel). By day 12 of the adherent culture, Ecad displayed a defined network formed by the protein localized to cell membranes (right Ecad panel). This was observed predominantly in colony regions containing clusters of cells with tight connections to each other. Expression of lst-1 was also evident at this developmental stage of the culture, appearing as an intracellular “dot” motif (Figure 4B, right bottom panel). This is in line with the previously published data showing that in mice, lst-1 mRNA expression is already present in fetal liver, and the protein is localized intracellularly before expressed on the plasma membrane. The latter is evident by postnatal day 29 [58] (for Review, see [59]). In a reference culture of primary hepatocytes, both types of lst-1 distribution were observed (Figure 4B, panel *e*).

In general, the above-described expression patterns of the proteins analysed were similar to those in primary hepatocytes cultured on collagen-coated plates (Figure 4B, panels *a–c, e*) or in liver tissue (panel *d*). Also, bi- and polynucleated cells were evident. These data support the conclusion that the spheroids exhibit advancing hepatic differentiation within outgrowing colonies, which correlated well with the decrease in the Afp gene activity.

Hence, the immunostaining analysis confirmed the capability of spheroids to give rise to advanced differentiated hepatocyte-like cell populations expressing hepatic proteins reflective of adult hepatocytes. This also strongly supports the hepatocyte precursor character of cells differentiated and selected in spheroid cultures, based on their qRT-PCR analysis.

### Glycogen storage and indocyanine green uptake in adherent cultures

**Figure 5:**
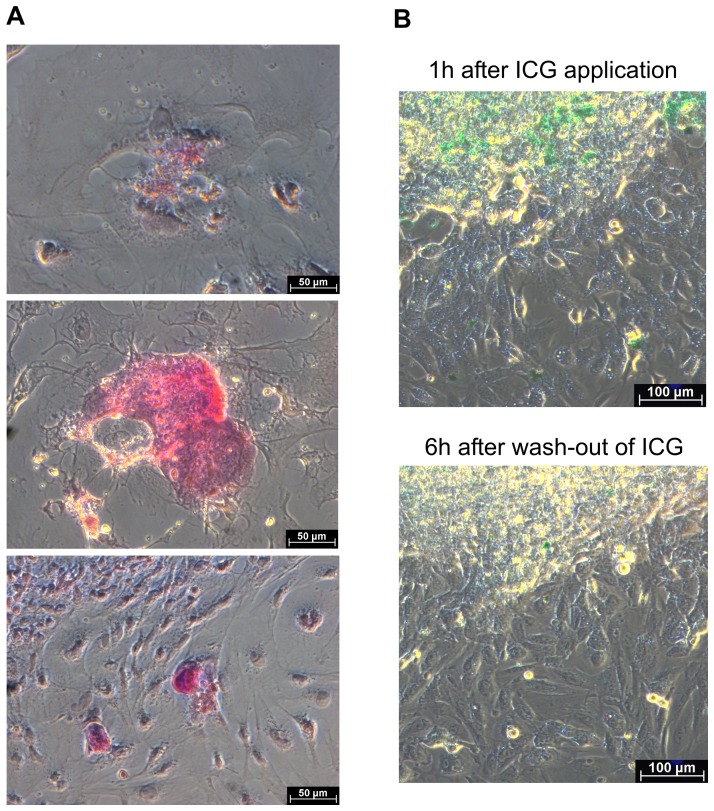
Glycogen storage and ICG up-take in spheroid-derived adherent colonies. (**A**) Glycogen-storing cells in a 12-day-old spheroid-derived adherent culture. (**B**) Cell cluster in the culture, reversibly up-taking ICG.

To evaluate further the spheroid-derived adherent cultures for their hepatic-like functionality, we stained 12-day-old cell colonies with Periodic acid-Schiff reagents to examine whether cells within the colonies stored glycogen. Glycogen-containing granules were detected not only in a number of single cells but also in cell groups or clusters (Figure 5A). In addition, cells or cell areas were able to reversibly take up ICG (Figure 5B). These findings are reflective of the functional features of native hepatocytes [60]–[63] and thus confirm appearance of cells featuring hepatocyte-like functionality in the adherent spheroid-derived cultures.

### Engraftment of spheroid cells in liver tissue

To investigate whether hepatocyte precursor cells selected in spheroid cultures are able to integrate into liver tissue, we transplanted 2-day-old spheroids into the partially hepatectomized mouse liver. One and 2 weeks after transplantation, animals were sacrificed and liver sections were evaluated for the presence of eGFP-expressing cells. Since the relatively small (about 30 kDa) eGFP molecules unspecifically translocate to some extent to the nucleus [64], [65], the engrafted cells could be particularly detected upon green fluorescence of their “thick” nuclei. In intensively fluorescent cells, also a thin layer of eGFP-positive cytoplasm was observed. Owing to the use of the HQ-Longpass Filterset for Enhanced-GFP, one could recognize eGFP-positive cells in the background of “snuff-coloured” autofluorescence of recipient liver tissue.

**Figure 6:**
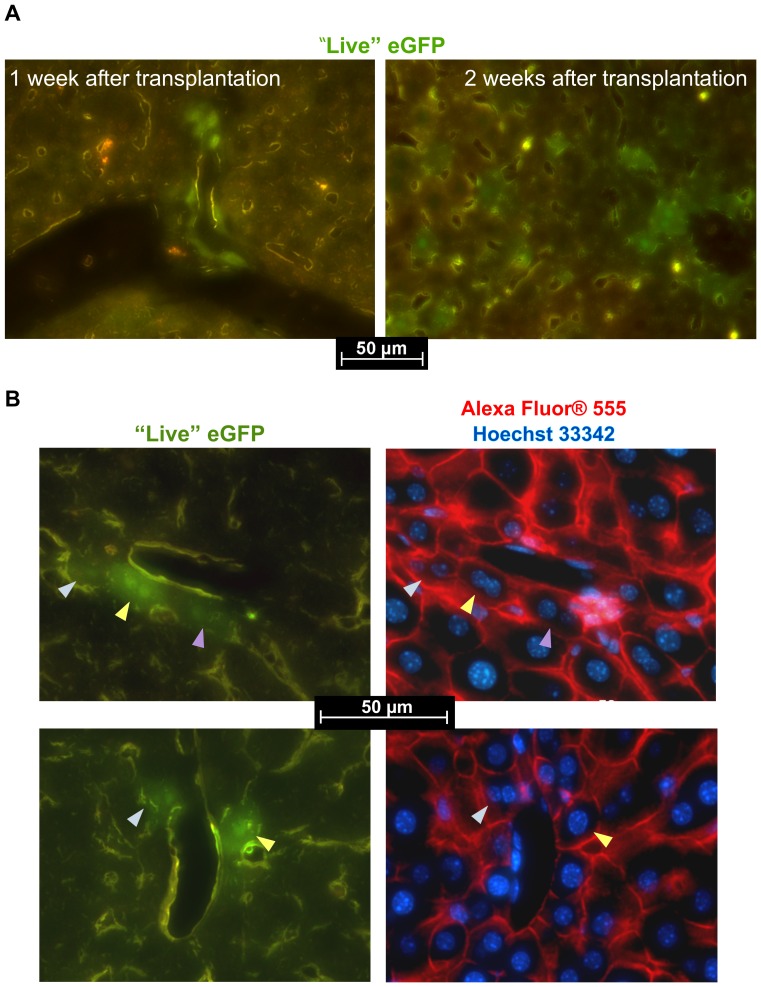
Engrafted spheroid-derived cells within recipient liver tissue. (**A**) eGFP-fluorescent cells and cell clusters observed in liver sections one and two weeks after spheroid transplantation. (**B**) Expression of Ecad in engrafted cells. “Live” eGFP and merged Alexa Fluor® 555/Hoechst 33342 images are shown.

At both above-mentioned time points, a number of single eGFP-expressing cells and even small clusters of such cells were detected in dozens of sections prepared from different parts of livers (data from three experiments, exemplified in Figure 6A). As can be recognized in the images, some of these cells were aligned along sinusoids or portal vein branches. Fluorescent immunostaining of liver sections for the epithelial-specific protein, Ecad, confirmed its presence in membranes of the engrafted cells, with a motif similar to that in other surrounding hepatocytes (Figure 6B). The Ecad expression pattern in eGFP-expressing cells, taken in relation to that in neighbouring hepatocytes, suggested that these cells integrated into surrounding tissue. As with native liver hepatocytes, one or two round nuclei were present in the engrafted cells. These findings pointed out that the integration of the spheroid-derived cells into recipient liver was accompanied by development of their hepatocyte-like features.

We did not detect any tumors in numerous liver sections observed microscopically one and two weeks after transplantation. But, as the 2-day-long exposure of spheroids to puromycin did not result in a complete elimination of undifferentiated ESCs (upon feeder test, 0.0005–0.003% ESCs remained in the culture), we believe that we would probably detect some tumors after a longer period of time. Further improvement of purification procedure is on our agenda for the next future.

Taken together, we can show that by applying the established dynamic EB and spheroid approaches, a population of hepatic lineage cells can be generated and selected, which displays both hepatic progenitor- and fetal hepatocyte-like features. This evidence was confirmed by the ability of the generated cell population to develop hepatic phenotype in both *in vitro* and *in vivo* conditions.

## Discussion

In this study we have established scalable suspension dynamic EB and spheroid cultures yielding hepatocyte-committed cells and, sequentially, a population of hepatocyte progenitor and(or) of fetal hepatocyte-like cells that feature the progressively developing hepatic gene expression phenotype and capacity to further differentiate into advanced hepatocyte-like cells and to integrate into recipient liver tissue.

There is increasing evidence that stem and progenitor cells are highly suitable for cell transplantation and tissue engineering of bio-artificial organs [66]–[68]. In addition, under appropriate culture conditions, these cells give rise to differentiated tissue-specific cell populations, which can be introduced as *in vitro* models for drug and toxicology testing [69] (for Review, see [70]). In particular, hepatocytes generated from ESCs could be routinely used to preclinical screen candidate substances for development of new therapeutic drugs [70]. This will have a prominent advantage over approaches implicating primary hepatocytes, immortalized cell lines, liver slices, and whole, perfused livers [71] (for Review, see [72]). Furthermore, *in vitro* produced ESC-derived hepatocyte precursors capable of efficient liver re-population because of their high proliferative capacity represent a promising transplantation material for prospective clinical trials. ESCs, in addition to their unlimited renewability and plasticity [73], are highly permissive in regards to genetic manipulations, allowing for their reliable stable transfection with transgenes possessing “live” monitoring and(or) selective markers under control of a tissue-specific gene regulatory sequence (promoter-enhancer) that becomes activated in the course of cell differentiation [70], [74]. This facilitates optimisation of culture protocols and also enables lineage selection of target cells - both attributes that are highly important for cell transplantation or establishment of *in vitro* pharmacological and toxicological screening systems.

Until now, attempts to maintain murine ESC-derived hepatocytes were mostly based on the use of two- or three-dimensional adhesive matrixes in a combination with appropriate media compositions or of co-culture systems promoting hepatic cell phenotype, with or without EB generation as a first step of the respective differentiation protocols (see, for example, [13]–[15], [75]–[84]). Although a lot of important data were obtained in these studies, providing evidence for the feasibility of the ESC system as a source of *in vitro* generated hepatic cells and thus promoting significant advances in this area, there are no reports of the successful establishment of highly purified scalable cultures of hepatocyte precursors or advanced hepatocyte-like cells.

Carpenedo and co-workers [85] demonstrated that a rotary suspension culture enhances the efficacy, output, and homogeneity of EB differentiation in relation to a static condition. Using transgenic ESC clones possessing the above-described reporter/selection gene construct, we established a dynamic spinner flask EB culture yielding hepatocyte lineage-committed cells, which are recognizable by their “live” eGFP fluorescence. Massive death of cells not expressing eGFP, concomitant with the expansion of eGFP-positive cells in the presence of puromycin, demonstrated efficiency of the drug pre-selection procedure used. To select and purify further the desired cell population, we next introduced a dynamic suspension spheroid culture as a “post-EB” three-dimensional mode. The spheroids were formed by eGFP-expressing cells separated from the puromycin-treated EBs. Analysis of RNA isolated from these spheroids revealed expression of hepatic genes. Up-regulation of *Alb*, *lst-1*, *Tat*, *Cyp7A1*, and *Mrp2* in the course of spheroid culture pointed to a progressive development of the hepatic gene expression phenotype in spheroid cells. Our conclusion that this process reflected the formation of a precursor/early hepatocyte-like cell population similar to liver fetal cells is supported by the fact that once placed into an adherent format, spheroids gave rise to expanding colonies, which expressed hepatic proteins with a pattern similar to that in primary hepatocytes, thus imitating the behaviour of the spheroid culture of fetal liver cells [32]. The ability of a number of cells within the adherent colonies to store glycogen and reversibly take up ICG suggested the appearance of functional hepatic cells. Since the expression of Afp declines to a low level in adult hepatocytes [86], the observed decrease in eGFP fluorescence (reflecting down-regulation of the Afp gene) along with the colony growth represents an additional indication of cell maturation.

It has been confirmed previously that the cellular morphological changes and cell-cell interactions caused by the spheroid formation are key factors promoting the expression of liver-specific functions in spheroid cultures generated from mouse fetal liver cells [32], [87]. In line with this, enhanced differentiation of adult bone marrow-derived stem cells to liver lineage in a three-dimensional aggregate culture in comparison to adherent monolayer cultures has been demonstrated [88]. Taking into account that no special medium or substances facilitating hepatic differentiation were applied in our experimental set, we assume that a similar mechanism significantly contributed to the liver fetal cell-like differentiation of spheroid cells. The three-dimensional spheroid cell “community” made obvious advances in terms of apparent activation of genes coding for proteins essential for important hepatocyte functions.

Further notable features of the spheroid cultures were their proliferative capacity and, as shown in experiments on spheroid transplantation, their ability to engraft into liver tissue and, similarly to native dividing hepatocytes in regenerating liver [89], to align along sinusoids. The latter is also in line with observations on engraftment of isolated mouse fetal liver cells in recipient liver [90], thus additionally proving the hepatocyte precursor/fetal hepatocyte-like character of the generated spheroid cultures. Similarly to transplanted fetal liver cells [90], the spheroid cells, which engrafted in hepatectomized liver where cells are stimulated to undergo mitosis [91], [92], most probably maintained proliferative capacity accompanied by *Afp* expression [89], [93]. This led to ongoing expression of eGFP under control of the Afp gene promoter.

Importantly, the established dynamic EB and spheroid procedures enabled a highly efficient lineage selection of target cells based on their progressive hepatocyte-specific differentiation.

Our preliminary data suggest a possibility to scale up the spheroid culture and completely eliminate undifferentiated ESCs, as well as to advance cell differentiation and maturation processes. It is also of importance to investigate the capability of spheroid cultures, which would have been completely purified from undifferentiated ESCs, to eventually re-populate injured liver without tumor formation over a long observation period. These experiments are in progress and their expected results are of a prospective clinical relevance because of the need of highly purified transplantable “young” hepatocyte-like cells that lack teratoma formation potential after their integration into the recipient liver.

In context of future prospects for cell therapy, for example for clinical transplantations, the transgenic cells might potentially possess a risk of triggering immune system response of a recipient by products of transgenes. But, since the selected spheroid cells are capable of further hepatic differentiation, the spheroid-derived grafts would most probably develop to advanced or mature hepatocyte cell populations. This would lead to a significant decrease in activity of the *Afp* promoter driving both eGFP and Pac cassettes [86], resulting in reduction or even prevention of the immunological response.

We believe that the dynamic culture system reported herein will contribute to the establishment of a large-scale production (e.g., in a bioreactor mode) of hepatocyte-like cultures, which could be used in clinical trials, as well as in *in vitro* screening procedures.

## Supporting Information

Table S1 List of genes analysed by real-time qRT-PCR.
(DOC)

Table S2 List of proteins analysed by fluorescent immunoassay.
(DOC)

## References

[1] Hewitt NJ, Lechon MJ, Houston JB, Hallifax D, Brown HS, et al (2007) Primary hepatocytes: current understanding of the regulation of metabolic enzymes and transporter proteins, and pharmaceutical practice for the use of hepatocytes in metabolism, enzyme induction, transporter, clearance, and hepatotoxicity studies. Drug Metab Rev 39: 159–234. 17364884 10.1080/03602530601093489

[2] Nussler A, Konig S, Ott M, Sokal E, Christ B, et al (2006) Present status and perspectives of cell-based therapies for liver diseases. J Hepatol 45: 144–159. 16730092 10.1016/j.jhep.2006.04.002

[3] Grossman M, Raper SE, Kozarsky K, Stein EA, Engelhardt JF, et al (1994) Successful ex vivo gene therapy directed to liver in a patient with familial hypercholesterolaemia. Nat Genet 4: 335–341. 10.1038/ng0494-335 8054972

[4] Lake JR (1998) Hepatocyte transplantation. N Engl J Med 338: 1464–1465. 10.1056/NEJM199805143382012 9580657

[5] Grompe M, Laconi E, Shafritz DA (1999) Principles of therapeutic liver repopulation. Semin Liver Dis 19: 7–14. 10349679 10.1055/s-2007-1007093

[6] Laconi E, Laconi S (2002) Principles of hepatocyte repopulation. Semin Cell Dev Biol 13: 433–438. 12468244 10.1016/s1084952102001313

[7] Dhawan A, Mitry RR, Hughes RD (2006) Hepatocyte transplantation for liver-based metabolic disorders. J Inherit Metab Dis 29: 431–435. 16763914 10.1007/s10545-006-0245-8

[8] Clayton DF, Darnell JE Jr (1983) Changes in liver-specific mRNA compared to common gene transcription during primary culture of mouse hepatocytes. Mol Cell Biol 3: 1552–1561. 6633533 10.1128/mcb.3.9.1552-1561.1983 PMC370008

[9] Bissell DM, Arenson DM, Maher JJ, Roll FJ (1987) Support of cultured hepatocytes by a laminin-rich gel. J Clin Invest 79: 801–812. 3546380 10.1172/JCI112887 PMC424203

[10] Godoy P, Hengstler JG, Ilkavets I, Meyer C, Bachmann A, et al (2009) Extracellular matrix modulates sensitivity of hepatocytes to fibroblastoid dedifferentiation and transforming growth factor beta-induced apoptosis. Hepatology 49: 2031–2043. 19274752 10.1002/hep.22880

[11] Zellmer S, Schmidt-Heck W, Godoy P, Weng H, Meyer C, et al (2010) Transcription factors ETF, E2F, and SP-1 are involved in cytokine-independent proliferation of murine hepatocytes. Hepatology 52: 2127–2136. 20979052 10.1002/hep.23930

[12] Hengstler JG, Brulport M, Schormann W, Bauer A, Hermes M, et al (2005) Generation of human hepatocytes by stem cell technology: definition of the hepatocyte. Expert Opin Drug Metab Toxicol 1: 61–74. 16922653 10.1517/17425255.1.1.61

[13] Dong XJ, Zhang GR, Zhou QJ, Pan RL, Chen Y, et al (2009) Direct hepatic differentiation of mouse embryonic stem cells induced by valproic acid and cytokines. World J Gastroenterol 15: 5165–5175. 19891015 10.3748/wjg.15.5165 PMC2773895

[14] Fukumitsu K, Ishii T, Yasuchika K, Amagai Y, Kawamura-Saito M, et al (2009) Establishment of a cell line derived from a mouse fetal liver that has the characteristic to promote the hepatic maturation of mouse embryonic stem cells by a coculture method. Tissue Eng Part A 15: 3847–3856. 19558317 10.1089/ten.TEA.2009.0357

[15] Liu T, Zhang S, Chen X, Li G, Wang Y (2010) Hepatic differentiation of mouse embryonic stem cells in three-dimensional polymer scaffolds. Tissue Eng Part A 16: 1115–1122. 19916802 10.1089/ten.TEA.2009.0391

[16] Baharvand H, Hashemi SM, Kazemi Ashtiani S, Farrokhi A (2006) Differentiation of human embryonic stem cells into hepatocytes in 2D and 3D culture systems in vitro. Int J Dev Biol 50: 645–652. 16892178 10.1387/ijdb.052072hb

[17] Basma H, Soto-Gutiérrez A, Yannam GR, Liu L, Ito R, et al (2009) Differentiation and transplantation of human embryonic stem cell-derived hepatocytes. Gastroenterology 136: 990–999. 19026649 10.1053/j.gastro.2008.10.047 PMC2732349

[18] Brolén G, Sivertsson L, Björquist P, Eriksson G, Ek M, et al (2010) Hepatocyte-like cells derived from human embryonic stem cells specifically via definitive endoderm and a progenitor stage. J Biotechnol 145: 284–294. 19932139 10.1016/j.jbiotec.2009.11.007

[19] Brulport M, Schormann W, Bauer A, Hermes M, Elsner C, et al (2007) Fate of extrahepatic human stem and precursor cells after transplantation into mouse livers. Hepatology 46: 861–870. 17668884 10.1002/hep.21745

[20] Erdö F, Buhrle C, Blunk J, Hoen M, Xia Y, et al (2003) Host-dependent tumorigenesis of embryonic stem cell transplantation in experimental stroke. J Cereb Blood Flow Metab 23: 780–785. 12843782 10.1097/01.WCB.0000071886.63724.FB

[21] Fujikawa T, Oh SH, Pi L, Hatch HM, Schupe T, et al (2005) Teratoma formation leads to failure of treatment for type I diabetes using embryonic stem cell-derived insulin-producing cells. Am J Pathol 166: 1781–1791. 15920163 10.1016/S0002-9440(10)62488-1 PMC1602425

[22] Duncan SA (2003) Mechanisms controlling early development of the liver. Mech Dev 120: 19–33. 12490293 10.1016/s0925-4773(02)00328-3

[23] Lemaigre F, Zaret KS (2004) Liver development update: new embryo models, cell lineage control, and morphogenesis. Curr Opin Genet Dev 14: 582–590. 15380251 10.1016/j.gde.2004.08.004

[24] Doetschman TC, Eistetter HR, Katz M, Schmidt W, Kemler R (1985) The in vitro development of blastocyst-derived embryonic stem cell lines: formation of visceral yolk sac, blood islands and myocardium. J Embryol Exp Morphol 87: 27–45. 3897439

[25] Shen MM, Leder P (1992) Leukemia inhibitory factor is expressed by the preimplantation uterus and selectively blocks primitive ectoderm formation in vitro. Proc Natl Acad Sci U S A 89: 8240–8244. 1518852 10.1073/pnas.89.17.8240 PMC49893

[26] Bielinska M, Wilson DB (1997) Induction of yolk sac endoderm in GATA-4-deficient embryoid bodies by retinoic acid. Mech Dev 65: 43–54. 9256344 10.1016/s0925-4773(97)00053-1

[27] Paparella M, Kolossov E, Fleischmann BK, Hescheler J, Bremer S (2002) The use of quantitative image analysis in the assessment of in vitro embryotoxicity endpoints based on a novel embryonic stem cell clone with endoderm-related GFP expression. Toxicol In Vitro 16: 589–597. 12206826 10.1016/s0887-2333(02)00052-8

[28] Choi D, Lee HJ, Jee S, Jin S, Koo SK, et al (2006) In vitro differentiation of mouse embryonic stem cells: Enrichment of endodermal cells in the embryoid body. Stem Cells 23: 817–827. 10.1634/stemcells.2004-0262 15917477

[29] Yin Y, Lim YK, Salto-Tellez M, Ng SC, Lin CS, et al (2002) AFP(+), ESC-derived cells engraft and differentiate into hepatocytes in vivo. Stem Cells 20: 338–346. 12110703 10.1634/stemcells.20-4-338

[30] Ishii T, Yasuchika K, Fujii H, Hoppo T, Baba S, et al (2005) In vitro differentiation and maturation of mouse embryonic stem cells into hepatocytes. Exp Cell Res 309: 68–77. 16009362 10.1016/j.yexcr.2005.05.028

[31] Drobinskaya I, Linn T, Šarić T, Bretzel RG, Bohlen H, et al (2008) Scalable selection of hepatocyte- and hepatocyte precursor-like cells from culture of differentiating transgenically modified murine embryonic stem cells. Stem Cells 26: 2245–2256. 18556507 10.1634/stemcells.2008-0387

[32] Tsuchiya A, Heike T, Fujino H, Shiota M, Umeda K, et al (2005) Long-term extensive expansion of mouse hepatic stem/progenitor cells in a novel serum-free culture system. Gastroenterology 128: 2089–2104. 15940640 10.1053/j.gastro.2005.03.030

[33] Livak KJ, Schmittgen TD (2001) Analysis of relative gene expression data using real-time quantitative PCR and the 2(-Delta Delta C(T)). Methods 25: 402–408. 11846609 10.1006/meth.2001.1262

[34] Kolossov E, Bostani T, Roell W, Breitbach M, Pillekamp F, et al (2006) Engraftment of engineered ES cell-derived cardiomyocytes but not BM cells restores contractile function to the infarcted myocardium. J Exp Med 203: 2315–2327. 16954371 10.1084/jem.20061469 PMC2118112

[35] Dziadek M (1978) Modulation of alphafetoprotein synthesis in the early postimplantation mouse embryo. J Embryol Exp Morphol 46: 135–146. 81255

[36] Dziadek MA, Andrews GK (1983) Tissue specificity of alpha-fetoprotein messenger RNA expression during mouse embryogenesis. EMBO J 2: 549–554. 6194986 10.1002/j.1460-2075.1983.tb01461.x PMC555059

[37] Liu H, Lin J, Roy K (2006) Effect of 3D scaffold and dynamic culture condition on the global gene expression profile of mouse embryonic stem cells. Biomaterials 27: 5978–5989. 16824594 10.1016/j.biomaterials.2006.05.053

[38] Xanthopoulos KG, Prezioso VR, Chen WS, Sladek FM, Cortese R, et al (1991) The different tissue transcription patterns of genes for HNF-1, C/EBP, HNF-3, and HNF-4, protein factors that govern liver-specific transcription. Proc Natl Acad Sci U S A 88: 3807–3811. 2023930 10.1073/pnas.88.9.3807 PMC51542

[39] Sasaki H, Hogan BL (1993) Differential expression of multiple fork head related genes during gastrulation and axial pattern formation in the mouse embryo. Development 118: 47–59. 8375339 10.1242/dev.118.1.47

[40] Monaghan AP, Kaestner KH, Grau E, Schütz G (1993) Postimplantation expression patterns indicate a role for the mouse forkhead/HNF-3 alpha, beta and gamma genes in determination of the definitive endoderm, chordamesoderm and neuroectoderm. Development 119: 567–578. 8187630 10.1242/dev.119.3.567

[41] Ang SL, Wierda A, Wong D, Stevens KA, Cascio S, et al (1999) The formation and maintenance of the definitive endoderm lineage in the mouse: involvement of HNF3/forkhead proteins. Development 119: 1301–1315. 10.1242/dev.119.4.1301 8306889

[42] Meehan RR, Barlow DP, Hill RE, Hogan BL, Hastie ND (1984) Pattern of serum protein gene expression in mouse visceral yolk sac and foetal liver. EMBO J 3: 1881–1885. 6479150 10.1002/j.1460-2075.1984.tb02062.x PMC557612

[43] Thomas T, Southwell BR, Schreiber G, Jaworowski A (1990) Plasma protein synthesis and secretion in the visceral yolk sac of the fetal rat: gene expression, protein synthesis and secretion. Placenta 11: 413–430. 1707170 10.1016/s0143-4004(05)80216-4

[44] Kakyo M, Unno M, Tokui T, Nakagomi R, Nishio T, et al (1999) Molecular characterization and functional regulation of a novel rat liver-specific organic anion transporter rlst-1. Gastroenterology 117: 770–775. 10500057 10.1016/s0016-5085(99)70333-1

[45] Lu H, Choudhuri S, Ogura K, Csanaky IL, Lei X, et al (2008) Characterization of organic anion transporting polypeptide 1b2-null mice: essential role in hepatic uptake/toxicity of phalloidin and microcystin-LR. Toxicol Sci 103: 35–45. 18296417 10.1093/toxsci/kfn038 PMC3180850

[46] Asahina K, Fujimori H, Shimizu-Saito K, Kumashiro Y, Okamura K, et al (2004) Expression of the liver-specific gene Cyp7a1 reveals hepatic differentiation in embryoid bodies derived from mouse embryonic stem cells. Genes Cells 9: 1297–1308. 15569160 10.1111/j.1365-2443.2004.00809.x

[47] Greengard O (1969) The hormonal regulation of enzymes in penatal and postnatal rat liver. Effects of adenosine 3′,5′-(cyclic)-monophosphate. Biochem J 115: 19–24. 4186680 10.1042/bj1150019 PMC1185063

[48] Gordon GJ, Coleman WB, Grisham JW (2000) Temporal analysis of hepatocyte differentiation by small hepatocyte-like progenitor cells during liver regeneration in retrorsine-exposed rats. Am J Pathol 157: 771–786. 10980117 10.1016/S0002-9440(10)64591-9 PMC1885692

[49] Nies AT, Keppler D (2007) The apical conjugate efflux pump ABCC2 (MRP2). Pflugers Arch 453: 643–659. 16847695 10.1007/s00424-006-0109-y

[50] Tamai M, Yamashita A, Tagawa YI (2011) Mitochondrial development of the in vitro hepatic organogenesis model with simultaneous cardiac mesoderm differentiation from murine induced pluripotent stem cells. J Biosci Bioeng 112: 495–500. 21816670 10.1016/j.jbiosc.2011.07.005

[51] Rogers J, Kalsheker N, Wallis S, Speer A, Coutelle CH, et al (1983) The isolation of a clone for human alpha 1-antitrypsin and the detection of alpha 1-antitrypsin in mRNA from liver and leukocytes. Biochem Biophys Res Commun 116: 375–382. 6606425 10.1016/0006-291x(83)90532-6

[52] Coakley RJ, Taggart C, O'Neill S, McElvaney NG (2001) Alpha1-antitrypsin deficiency: biological answers to clinical questions. Am J Med Sci 321: 33–41. 11202478 10.1097/00000441-200101000-00006

[53] Franke WW, Schmid E, Kartenbeck J, Mayer D, Hacker HJ, et al (1979) Characterization of the intermediate-sized filaments in liver cells by immunofluorescence and electron microscopy. Biol Cell 34: 99–110.

[54] Zatloukal K, Stumptner C, Lehner M, Denk H, Baribault H, et al (2000) Cytokeratin 8 protects from hepatotoxicity, and its ratio to cytokeratin 18 determines the ability of hepatocytes to form Mallory bodies. Am J Pathol 156: 1263–1274. 10751352 10.1016/S0002-9440(10)64997-8 PMC1876873

[55] Vestweber D, Kemler R (1984) Some structural and functional aspects of the cell adhesion molecule uvomorulin. Cell Differ 15: 269–273. 6336035 10.1016/0045-6039(84)90084-8

[56] Butz S, Larue L (1995) Expression of Catenins during mouse embryonic development and in adult tissues. Cell Adhes Commun 3: 337–352. 8821035 10.3109/15419069509081018

[57] Ueberham E, Aigner T, Ueberham U, Gebhardt R (2007) E-cadherin as a reliable cell surface marker for the identification of liver specific stem cells. J Mol Histol 38: 359–368. 17605082 10.1007/s10735-007-9098-1

[58] Gao B, St Pierre MV, Stieger B, Meier PJ (2004) Differential expression of bile salt and organic anion transporters in developing rat liver. J Hepatol 41: 201–208. 15288467 10.1016/j.jhep.2004.04.029

[59] Myllynen P, Immonen E, Kummu M, Vähäkangas K (2009) Developmental expression of drug metabolizing enzymes and transporter proteins in human placenta and fetal tissues. Expert Opin Drug Metab Toxicol 5: 1483–1499. 19785513 10.1517/17425250903304049

[60] Jungermann K, Heilbronn R, Katz N, Sasse D (1982) The glucose/glucose-6-phosphate cycle in the periportal and perivenous zone of rat liver. Eur J Biochem 123: 429–436. 6281009 10.1111/j.1432-1033.1982.tb19786.x

[61] Probst I, Schwartz P, Jungermann K (1982) Induction in primary culture of ‘gluconeogenic’ and ‘glycolytic’ hepatocytes resembling periportal and perivenous cells. Eur J Biochem 126: 271–278. 6751822 10.1111/j.1432-1033.1982.tb06775.x

[62] Berk PD, Stremmel W (1986) Hepatocellular uptake of organic anions. Prog Liver Dis 8: 125–144. 3520658

[63] Meijer DKF, Weert B, Vermeer GA (1988) Pharmacokinetics of biliary excretion in man. VI. Indocyanine green. Eur J Clin Pharmacol 35: 295–303. 3181282 10.1007/BF00558268

[64] Grebenok RJ, Pierson E, Lambert GM, Gong FC, Afonso CL, et al (1997) Green-fluorescent protein fusions for efficient characterization of nuclear targeting. Plant J 11: 573–586. 9107043 10.1046/j.1365-313x.1997.11030573.x

[65] Seibel NM, Eljouni J, Nalaskowski MM, Hampe W (2007) Nuclear localization of enhanced green fluorescent protein homomultimers. Anal Biochem 368: 95–99. 17586454 10.1016/j.ab.2007.05.025

[66] Kubota H, Reid LM (2000) Clonogenic hepatoblasts, common precursors for hepatocytic and biliary lineages, are lacking classical major histocompatibility complex class I antigen. Proc Natl Acad Sci U S A 97: 12132–12137. 11050242 10.1073/pnas.97.22.12132 PMC17306

[67] Weissman IL (2000) Translating stem and progenitor cell biology to the clinic: barriers and opportunities. Science 287: 1442–1446. 10688785 10.1126/science.287.5457.1442

[68] Oertel M, Menthena A, Dabeva MD, Shafritz DA (2006) Cell competition leads to a high level of normal liver reconstitution by transplanted fetal liver stem/progenitor cells. Gastroenterology 130: 507–520. 16472603 10.1053/j.gastro.2005.10.049

[69] Seiler A, Visan A, Buesen R, Genschow E, Spielmann H (2004) Improvement of an in vitro stem cell assay for developmental toxicity: The use of molecular endpoints in the embryonic stem cell test. Reprod Toxicol 18: 231–240. 15019721 10.1016/j.reprotox.2003.10.015

[70] Davila JC, Cezar GG, Thiede M, Strom S, Miki T, et al (2004) Use and application of stem cells in toxicology. Toxicol Sci 79: 214–223. 15014205 10.1093/toxsci/kfh100

[71] Lee HJ, Cha KE, Hwang SG, Kim JK, Kim GJ (2011) In vitro screening system for hepatotoxicity: comparison of bone-marrow-derived mesenchymal stem cells and Placenta-derived stem cells. J Cell Biochem 112: 49–58. 20524208 10.1002/jcb.22728

[72] Farkas D, Tannenbaum SR (2005) In vitro methods to study chemically-induced hepatotoxicity: a literature review. Curr Drug Metab 6: 111–125. 15853763 10.2174/1389200053586118

[73] Edwards RG (2004) Stem cells today: A. Origin and potential of embryo stem cells. Reprod Biomed Online 8: 275–306. 15038895 10.1016/s1472-6483(10)60910-8

[74] Lai Y, Drobinskaya I, Kolossov E, Chen C, Linn T (2008) Genetic modification of cells for transplantation. Adv Drug Deliv Rev 60: 146–159. 18037530 10.1016/j.addr.2007.08.039

[75] Hamazaki T, Iiboshi Y, Oka M, Papst PJ, Meacham AM, et al (2001) Hepatic maturation in differentiating embryonic stem cells in vitro. FEBS Lett 497: 15–19. 11376655 10.1016/s0014-5793(01)02423-1

[76] Yamada T, Yoshikawa M, Kanda S, Kato Y, Nakajima Y, et al (2002) In vitro differentiation of embryonic stem cells into hepatocyte-like cells identified by cellular uptake of indocyanine green. Stem Cells 20: 146–154. 11897871 10.1634/stemcells.20-2-146

[77] Kanda S, Shiroi A, Ouji Y, Birumachi J, Ueda S, et al (2003) In vitro differentiation of hepatocyte-like cells from embryonic stem cells promoted by gene transfer of hepatocyte nuclear factor 3 beta. Hepatol Res 26: 225–231. 12850695 10.1016/s1386-6346(03)00114-1

[78] Kania G, Blyszczuk P, Jochheim A, Ott M, Wobus AM (2004) Generation of glycogen-and albumin-producing hepatocyte-like cells from embryonic stem cells. Biol Chem 385: 943–953. 15551869 10.1515/BC.2004.123

[79] Imamura T, Cui L, Teng R, Johkura K, Okouchi Y, et al (2004) Embryonic stem cell-derived embryoid bodies in three-dimensional culture system form hepatocyte-like cells in vitro and in vivo. Tissue Eng 10: 1716–1724. 15684680 10.1089/ten.2004.10.1716

[80] Teratani T, Yamamoto H, Aoyagi K, Sasaki H, Asari A, et al (2005) Direct hepatic fate specification from mouse embryonic stem cells. Hepatology 41: 836–846. 15742390 10.1002/hep.20629

[81] Tsutsui M, Ogawa S, Inada Y, Tomioka E, Kamiyoshi A, et al (2006) Characterization of cytochrome P450 expression in murine embryonic stem cell-derived hepatic tissue system. Drug Metab Dispos 34: 696–701. 16415121 10.1124/dmd.105.007674

[82] Fang S, Qiu YD, Mao L, Shi XL, Yu DC, et al (2007) Differentiation of embryoid-body cells derived from embryonic stem cells into hepatocytes in alginate microbeads in vitro. Acta Pharmacol Sin 28: 1924–1930. 18031606 10.1111/j.1745-7254.2007.00713.x

[83] Moore RN, Dasgupta A, Rajaei N, Yarmush ML, Toner M, et al (2008) Enhanced differentiation of embryonic stem cells using co-cultivation with hepatocytes. Biotechnol Bioeng 101: 1332–1343. 18571804 10.1002/bit.21987 PMC4492440

[84] Li Y, Kang X, Guo K, Li X, Gao D, et al (2009) Proteome alteration of early-stage differentiation of mouse embryonic stem cells into hepatocyte-like cells. Electrophoresis 30: 1431–1440. 19424999 10.1002/elps.200800836

[85] Carpenedo RL, Sargent CY, McDevitt TC (2007) Rotary suspension culture enhances the efficiency, yield, and homogeneity of embryoid body differentiation. Stem Cells 25: 2224–2234. 17585171 10.1634/stemcells.2006-0523

[86] Jin DK, Vacher J, Feuerman MH (1998) alpha-Fetoprotein gene sequences mediating Afr2 regulation during liver regeneration. Proc Natl Acad Sci U S A 95: 8767–8772. 9671753 10.1073/pnas.95.15.8767 PMC21151

[87] Hamamoto R, Yamada K, Kamihira M, Iijima S (1998) Differentiation and proliferation of primary rat hepatocytes cultured as spheroids. J Biochem 124: 972–979. 9792921 10.1093/oxfordjournals.jbchem.a022215

[88] Subramanian K, Owens DJ, O'Brien TD, Verfaillie CM, Hu WS (2011) Enhanced differentiation of adult bone marrow-derived stem cells to liver lineage in aggregate culture. Tissue Eng Part A 17: 2331–2341. 21548835 10.1089/ten.tea.2010.0667 PMC3161102

[89] Hoehme S, Brulport M, Bauer A, Bedawy E, Schormann W, et al (2010) Prediction and validation of cell alignment along microvessels as order principle to restore tissue architecture in liver regeneration. Proc Natl Acad Sci U S A 107: 10371–10376. 20484673 10.1073/pnas.0909374107 PMC2890786

[90] Nierhoff D, Ogawa A, Oertel M, Chen YQ, Shafritz DA (2005) Purification and characterization of mouse fetal liver epithelial cells with high in vivo repopulation capacity. Hepatology 42: 130–139. 15895427 10.1002/hep.20735

[91] Alison MR, Golding MHC, Sarraf CE (1996) Pluripotent liver stem cells: facultative stem cells located in the biliary tree. Cell Prolif 29: 373–402. 8883463 10.1111/j.1365-2184.1996.tb00982.x

[92] Michalopoulos GK, DeFrances MC (1997) Liver regeneration. Science 276: 60–66. 9082986 10.1126/science.276.5309.60

[93] Sell S, Becke FF, Leffert HL, Watabe L (1976) Expression of an oncodevelopmental gene product (alpha-fetoprotein) during fetal development and adult oncogenesis. Cancer Res 36: 4239–4249. 61804

