# Supplementary material for: Differentiation and Selection of Hepatocyte Precursors in Suspension Spheroid Culture of Transgenic Murine Embryonic Stem Cells
Source: PLoS One. 2012 Sep 24;7(9):e44912. doi: 10.1371/journal.pone.0044912 (PMC3454367; doi:10.1371/journal.pone.0044912)
Supplement: Table S1 — List of genes analysed by real-time qRT-PCR. (DOC) [file pone.0044912.s001.doc]

Supplementary Table 1. List of genes analysed by real-time qRT-PCR.

| **Gene name** | **Other designations** | **Gene symbol** | **Gene aliases** | **Entrez Gen ID** | **TaqMan® Gene Expression Assay** |
| --- | --- | --- | --- | --- | --- |
| forkhead box A2 | HNF-3-beta, C; forkhead box factor | Foxa2 | HNF3-beta, Hnf3b, | 15376 | Mm00839704_mH |
|  | protein A2; hepatocyte nuclear |  | HNF3beta, Hnf-3b, |  |  |
|  | 3 beta (winged helix transcription |  | RP23-207P16.2, Tcf- |  |  |
|  | factor) |  | 3b, Tcf3b |  |  |
| alpha-fetoprotein | alpha-1-fetoprotein; alpha- | Afp | - | 11576 | Mm00431715_m1* |
|  | fetoglobulin; alpha-foetoprotein |  |  |  |  |
| albumin | albumin 1; serum albumin | Alb | Alb-1, Alb1 | 11657 | Mm00802090_m1* |
| cytochrome P450, | CYPVII; cholesterol 7 alpha | Cyp7A1 | RP23-89L15.2 | 13122 | Mm00484152_m1* |
| family 7, subfamily a, | hydroxylase; cholesterol 7-alpha- |  |  |  |  |
| polypeptide 1 | hydroxylase; cholesterol 7-alpha- |  |  |  |  |
|  | monooxygenase |  |  |  |  |
| liver-specific organic | solute carrier organic anion | lst-1 | Slco1b2, OATP-C, | 28253 | Mm00451510_m1* |
| anion transporter 1 | transporter family, member 1b2 |  | 7330442B20Rik, |  |  |
|  |  |  | OATP2, Oatp1b2, |  |  |
|  |  |  | Oatp4, Slc21a10, |  |  |
|  |  |  | Slc21a6, mlst-1 |  |  |
| tyrosine | L-tyrosine:2-oxoglutarate | Tat | MGC37772, | 234724 | Mm01244282_m1* |
| aminotransferase | aminotransferase; tyrosine |  | MGC37789, |  |  |
|  | transaminase |  | MGC37790, |  |  |
|  |  |  | MGC37819, |  |  |
|  |  |  | MGC37828, |  |  |
|  |  |  | MGC37842 |  |  |
| multidrug resistance- | ATP-binding cassette, sub-family C | Mrp2 | Abcc2, AI173996, | 12780 | Mm00496899_m1* |
| associated protein 2 | anion transporter 1 (CFTR/MRP), member 2; canalicular multispecific |  | Abc30, Cmoat, cMRP |  |  |
|  | organic anion transporter 1; |  |  |  |  |
|  | multidrug resistance protein 2 |  |  |  |  |
| hypoxanthine guanine | HGPRTase; HPRT B; | Hprt | C81579, HPGRT, | 15452 | Mm00446968_m1* |
| phosphoribosyl | hypoxanthine guanine |  | Hprt1, MGC103149, |  |  |
| transferase | phosphoribosyl transferase 1; |  | RP23-173F3.3 |  |  |
|  | hypoxanthine-guanine |  |  |  |  |
|  | phosphoribosyltransferase |  |  |  |  |
| TATA box binding | TATA sequence-binding protein; | Tbp | GTF2D1, Gtf2d, | 21374 | Mm00446973_m1* |
| protein | TATA-binding factor; TATA- |  | SCA17, TFIID |  |  |
|  | binding protein; TATA-box factor; |  |  |  |  |
|  | TATA-box-binding protein |  |  |  |  |
