# Supplementary material for: Differentiation and Selection of Hepatocyte Precursors in Suspension Spheroid Culture of Transgenic Murine Embryonic Stem Cells
Source: PLoS One. 2012 Sep 24;7(9):e44912. doi: 10.1371/journal.pone.0044912 (PMC3454367; doi:10.1371/journal.pone.0044912)
Supplement: Table S2 — List of proteins analysed by fluorescent immunoassay. (DOC) [file pone.0044912.s002.doc]

Supplementary Table 2. List of proteins analysed by fluorescent immunoassay.

| **Protein name** | **Other designations** | **Protein symbol** | **Protein aliases** | **UniProt ID** |
| --- | --- | --- | --- | --- |
| Albumin | Serum albumin | Alb | Alb-1, Alb1 | **P07724** |
| Alpha-1-antitrypsin | Alpha-1 protease inhibitor 1, | Aat | Serpin A1a | **P07758** |
|  | Alpha-1-antiproteinase, |  |  |  |
|  | Serine protease inhibitor 1-1, |  |  |  |
|  | Serine protease inhibitor A1a |  |  |  |
| Cytokeratin-18 | Keratin, type I cytoskeletal 18, | CK-18 | Keratin D, K18 | **P05784** |
|  | Cytokeratin endo B, Keratin-18 |  |  |  |
| E-cadherin | Cadherin 1, ARC-1, Epithelial | Ecad | Cdh1, E-Cad | **P09803** |
|  | cadherin, Uvomorulin |  |  |  |
| Liver-specific organic | Solute carrier organic anion | lst-1 | Slco1b2, Oatp1b2, | Q9JJL3 |
| anion transporter 1 | transporter family member 1B2**,** |  | Slc21a10, Slc21a6, |  |
|  | Solute carrier family 21 member 10 |  | mlst-1 |  |
